# Strategy of Hepatic Metabolic Defects Induced by *beclin1* Heterozygosity in Adult Zebrafish

**DOI:** 10.3390/ijms21041533

**Published:** 2020-02-24

**Authors:** Suzan Attia Mawed, Yan He, Jin Zhang, Jie Mei

**Affiliations:** 1 College of Fisheries, Huazhong Agricultural University, Wuhan 430070, China; zoologist_zoologist@yahoo.com (S.A.M.); jzhang295411@163.com (J.Z.); 2 Zoology Department, Faculty of Science, Zagazig University, Zagazig 44519, Egypt

**Keywords:** autophagy, *beclin1*, liver metabolism, apoptosis, PI3K/AKT

## Abstract

Hepatic disorders have been increasing in recent years because of high carbohydrate diets. Hepatocytes depend mainly on the basal autophagy to maintain hepatic glucose/lipid homeostasis in mammals. However, the regulatory mechanisms of autophagy in hepatic energy metabolism are still unknown in fish species. Accordingly, mutant zebrafish lines of autophagy-related genes *beclin1* and *atg7* were generated by CRISPR/Cas9 gene-editing technology. Interestingly, unlike *atg7^+/−^*, male *beclin1^+/−^* zebrafish displayed liver defects in the morphology and histology, including abnormal hepatocyte proliferation, hemorrhagic and inflammatory phenotypes. A significant decrease in hepatocyte glycogen and an increase in hepatocyte lipids were detected in the histological assay that coincidence with the hepatic gene expression. Meanwhile, loss of heterozygosity for *beclin1* creates a suitable microenvironment for hepatic tumorigenesis via phosphorylation of Akt kinase, which in turn affects liver autophagy. The reduction in autophagy activity in male *beclin1^+/−^* liver leads to a disturbance in the glucose/lipid metabolism and negatively regulates apoptosis accompanied by the induction of cellular proliferation and acute inflammatory response. Our findings highlight an important role of *beclin1* in zebrafish liver development and energy metabolism, suggesting the crucial role of autophagy in maintaining homeostasis of the nutrient metabolism in fish species.

## 1. Introduction

The liver plays a crucial role in governing body energy, especially in glucose and fatty acid metabolism. After feeding, glucose is synthesized into glycogen or converted into fatty acids or amino acids in the liver. Fatty acids generate triacylglycerol (TAG) that can be stored in the hepatocytes. During fasting, TAG is released from the liver into the circulation, and it is metabolized into non-esterified fatty acids (NEFAs) and glycerol via lipolysis. Hence, both gluconeogenesis and fatty acid β-oxidation processes take place in the liver to release energy [1]. Accordingly, liver dysfunction usually leads to the disturbance of glucose and lipid metabolism, which affects the whole body’s energy and causes abnormal development.

Due to the high biosynthetic activity and important role of energy storage in the liver, hepatocytes, particularly, depend on basal autophagy that is the main way to sequester and degrade their intracellular contents and maintain energy balance in hepatocytes [2,3]. Autophagy is a lysosomal pathway for the degradation of long-lived proteins and damaged organelles [2,3,4], which is the major cellular route for maintaining cellular homeostasis and metabolism [5,6]. In mammals, impaired autophagy signaling is correlated with glucose and lipid metabolic disorders [4,5,6]. In Nile tilapia, body growth was inhibited by chloroquine, an inhibitor of autophagy, leading to increased glycolysis and fat accumulation accompanied by high inflammatory response [7]. However, the regulatory mechanisms of autophagy in the hepatic energy metabolism are still obscure in fish species.

Until now, more than 36 autophagy-related genes (Atgs) have been revealed to contribute to the organization of autophagy machinery, including two crucial genes *beclin1* and *atg7* [8]. *Beclin1* governs the initiation of the autophagy process by regulating the PI3K/AKT/mTOR pathway, an intracellular signaling pathway important in regulating the cell cycle [9], while atg7 is involved in the elongation of the autophagosomes membrane [10]. Beclin1 has been reported to be a haploinsufficient tumor suppressor gene [11], and monoallelic deletion is sufficient to promote tumorigenesis in the ovary [12,13], breast [14,15], prostate [16], and liver [11,17,18]. However, the involved molecular mechanisms are still poorly understood [19].

The phosphoinositide 3-kinase (PI3K) and its downstream kinases, such as AKT and mTOR, modulate numerous programmed signaling pathways involved in cell survival and cancer progression [20,21]. The synergistic combination of programmed cell pathways, including autophagy, apoptosis, and necrosis, may decide the fate of the cells [22]. Briefly, autophagy and apoptosis are vital catabolic pathways and are essential for normal cellular differentiation and growth [23,24]. In contrast, necrosis is a largely unregulated type of cell death that starts with uncontrolled cellular proliferation and ends by necroptosis that also leads to death [25]. Hence, when autophagy or apoptosis are blocked, the cell may still die via another biological way, and the disturbance of both pathways might lead to necrosis [26,27].

Some studies indicated that PI3K/AKT activity was elevated in the cells harboring high levels of tP53 mutant protein [28,29,30], and more than half of the solid tumors harbor mutated tP53 protein that suppresses autophagy and ceases cancer cell apoptosis [31,32,33]. On the other side, it has been reported that suppression of autophagy by PI3K/AKT activation accelerates tumor growth due to inflammation [34,35]. Interleukin-6, a family of cytokinesis, is usually associated with inflammation during carcinogenesis and has been reported to inhibit or delay apoptosis [36,37,38]. IL-6 is considered as a malevolent player that promotes tumor initiation and macrophages infiltration and is found to be closely related to STAT3 (Signal Transducers and Activators of Transcription-3) [38,39,40]. Moreover, overexpression of IL-6 promoted cell transformation by inhibiting autophagy [41,42]. Zebrafish (*Danio rerio*) is a valuable model to study human diseases, including liver tumors [43,44]. In the present study, the zebrafish mutant line of *beclin1* was constructed to assess the relationship between autophagy and hepatic metabolic disorder and defective development.

## 2. Results

### 2.1. CRISPR/Cas9-Mediated Targeted Mutagenesis of atg7 and beclin1 in Zebrafish

**Figure 1:**
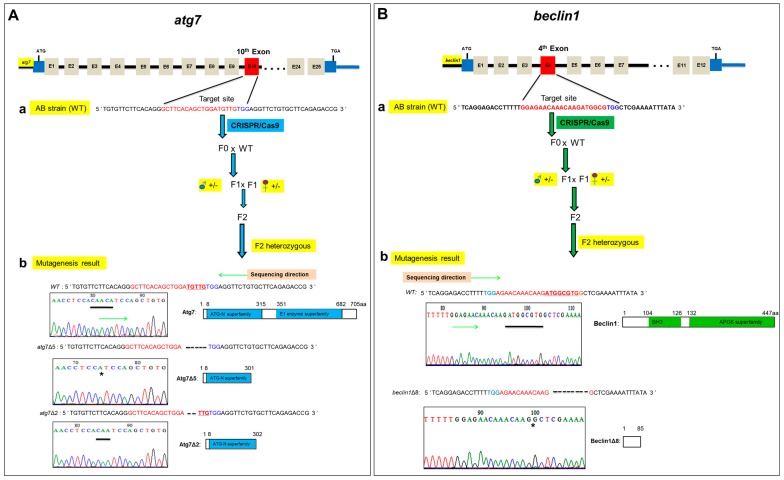
Generation of *atg7* and *beclin1* zebrafish mutants. (**A**) The procedure of *atg7* targeted mutagenesis. (**a**) Schematic representation of the zebrafish *atg7* target site and generation mating between *atg7* F1 heterozygous male (blue circle) and female (red circle) to produce F2 generation. The thin line and grey boxes represent the introns and exons, respectively, and the red box represents the target 10th exon. The sgRNA target sequence is shown in red, followed by a PAM sequence “TGG” shown in blue. (**b**) Genotyping and illustration of the deduced protein structure of wild-type *atg7* and two mutated *atg7*. The black bar indicates the target sequence of a *atg7* wild type (*n* = 5 bp) in the target site and the black star refers to the deletion part in mutants. (**B**) The procedure of *beclin1* targeted mutagenesis. (**a**) Schematic representation of the zebrafish *beclin1* target site and generation mating between *beclin1* F1 heterozygous male (blue circle) and female (red circle) to produce F2 generation. The red box represents the target 4th exon. The sgRNA target sequence is shown in red, followed by a PAM sequence “TGG” shown in blue. (**b**) Genotyping and illustration of the deduced protein structure of wild-type *beclin1* and the mutated *beclin1*. The black bar indicates the target sequence of a *beclin1* wild type (*n* = 8 bp) in the target site and the black star refers to the deletion part in mutants.

*Atg7* and *beclin1* mutant lines were generated using CRISPR/Cas9 gene-editing technology in zebrafish. Briefly, the sgRNA targeting sites were chosen in the 10th and 4th exons of *atg7* and *beclin1*, respectively. After microinjection of Cas9 and gRNA mRNAs into 1-cell zebrafish embryos, the growing embryos were termed as F0 generation. The chimera F0 mated with wild-type (WT) zebrafish to obtain the F1 heterozygous generation, from which we obtained F2 by mating heterozygous males and females (Figure 1Aa,Ba). To verify the specificity of *atg7* and *beclin1* heterozygous target sites, the PCR products obtained from the heterozygous F1 DNA were purified and inserted into the PMD™–18T vector for sequencing. Finally, two *atg7* mutant lines with 5-bp deletion (*atg7*Δ5) and 2-bp deletion (*atg7*Δ2) were produced, respectively (Figure 1Ab). Zebrafish Atg7 protein consists of two functional domains, including the ATG-N superfamily and E1 enzyme superfamily, and the deletions in the Atg7Δ5 and Atg7Δ2 resulted in a frame-shift caused by a premature stop codon. On the other side, one *beclin1* mutant line was established, which contained 8-bp deletion, named *beclin1*Δ8 (Figure 1Bb). Beclin1 protein consists of two functional domains, including BH3 and APG6 superfamily, and the deletions in Beclin1Δ8 resulted in a frame-shift caused by a premature stop codon in the mutant line.

### 2.2. Beclin1 Heterozygosity Affects Liver Histology and Causes High Mortality Rate in Male Fish

**Figure 2:**
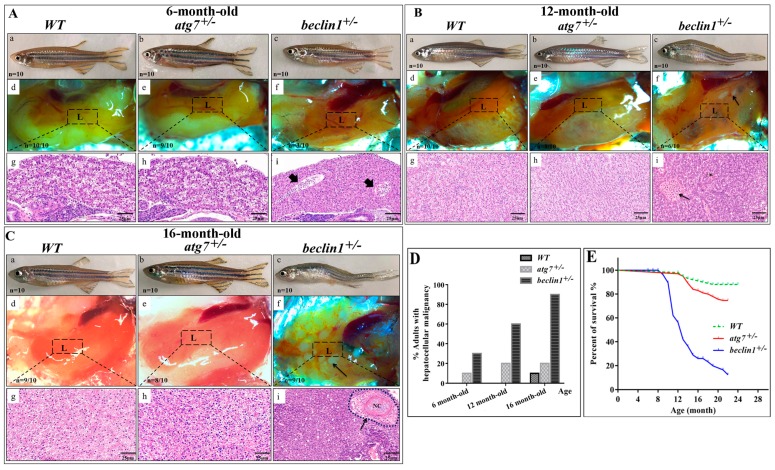
*Beclin1* heterozygosity leads to liver disorders in adult males. (**A**) (**a**–**c**) Representative pictures of 6-month-old male zebrafish. (**d**–**f**) The liver phenotypes in dissected males. (**g**–**i**) hematoxylin and eosin (H&E) histological staining of zebrafish livers and the beginning of bile ductus sequestration (arrowheads). (**B**) (**a**–**c**) Representative pictures of 12-month-old male zebrafish. *beclin1^+/−^* has enlarged the belly and curved the body. (**d**–**f**) The liver phenotypes in dissected males. Hepatic ulcers (black arrow) were observed in the *beclin1^+/−^* males. (**g**–**i**) H&E histological assay showing the tumor cells cords and bile sequestrations (black arrow) in *beclin1^+/−^*. (**C**) (**a**–**c**) Representative pictures of 16-month-old male zebrafish. (**d**–**f**) The liver phenotypes in dissected males. *beclin1^+/−^* liver had a hemorrhagic and necrotic appearance distributed on the inferior surface of the liver (black arrow). (**g**–**i**) H&E histological assay showing the development of confocal necrosis at 16-month-old male *beclin1^+/−^* (black arrow). NC: Necrosis. (**D**) Prevalence of microscopic hepatocellular malignancy. *n* = 10 for each experimental group. (**E**) Meire Kaplan graphs depicting the survival rate of the three reared strains. *n* = 50 for each experimental group. *Beclin1^+/−^* exhibited lower survival rate than WT at 12- and 16-month-old respectively (*p* < 0.0001). L: liver, the dotted frame represents liver histology in the underlined pictures.

WT and both reared heterozygous strains from F2 generation grew normally and did not manifest any distinct phenotype before 6-month-old, whereas the homozygous mutants of *atg7* and *beclin1* died at the larval stage. On dissection, the liver appeared normal, while histological observation showed the beginning of alternation in the hepatic parenchyma accompanied by a little proliferation and bile sequestration in some *beclin1^+/−^* males (Figure 2Aa–i). At 12-month-old, *beclin1* heterozygous exhibited curved bodies with enlarged belly and the liver covered most of the abdominal cavity where small white ulcers developed on its dorsal side (Figure 2Ba–f). Histological analysis revealed abnormal hepatocyte proliferation that appeared in irregular cords in the *beclin1^+/−^* fish, unlike the WT and *atg7^+/−^* individuals (Figure 2Bg–i). At 16-months-old, *beclin1^+/−^* males became feebler with exhausted energy and obvious hepatic tumors, hemorrhagic, and inflammatory appearance (Figure 2Ca–f). Hepatic tissue of *beclin1^+/−^* exhibited heavily cellular condensation with pyknosis and mature focal necrosis could be observed (Figure 2Cg–i). After macroscopic observations and histological analysis in 6, 12, and 16-month-old *beclin1^+/−^* males, we could determine the microscopic tumorigenesis distributed in the liver. At 6-month-old, liver of *beclin1^+/−^* exhibited a little increase in cellular proliferation. At 12-month-old, hepatic tissues were modified into diffused cords that increased by 16-month-old with solid necrosis in about 90% of the *beclin1^+/−^* zebrafish (Figure 2D). Almost no mortality occurred before 6-month-old in the *beclin1^+/−^* zebrafish, whereas 40% and 75% of *beclin1^+/−^* males died at 12 and 16-month-old, respectively. On the other side, WT and *atg7^+/−^* males did not exhibit changes during the developing time, and little mortality has been recorded (Figure 2E).

### 2.3. Heterozygosity of beclin1 Alters Hepatic Energy Metabolism in Male Zebrafish

**Figure 3:**
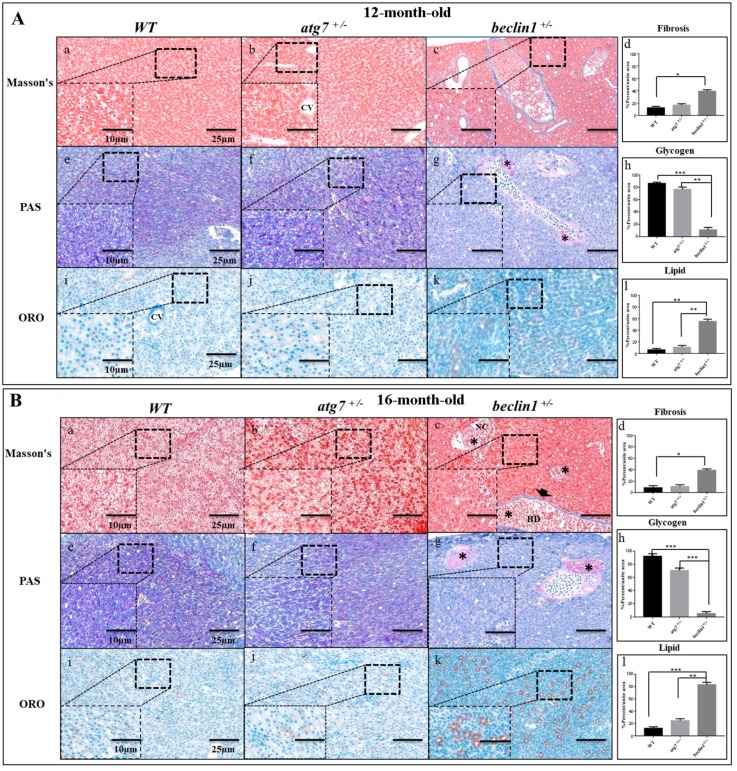
Defects of hepatic energy metabolism in male *Beclin1^+/−^* zebrafish. (**A**) Histological assessment of hepatocytes in 12-month-old male zebrafish. (**a**–**d**) Representative photomicrographs of Masson’s trichrome staining and the quantification of fibrosis. (**e**–**h**) Representative photomicrographs of Periodic Acid-Schiff (PAS) staining and glycogen content showing that glycogen packed in the hepatocytes of wild type (WT) and *atg7^+/−^* and lacked in *beclin1^+/−^* zebrafish; notice the beginning of hepatocellular malignancy (black star) around bile ducts. (**i**–**l**) Oil Red O (ORO) staining and lipid quantification revealing the alternations in fat droplet formation in *beclin1^+/−^* hepatocytes. (**B**) Histological assessment of hepatocytes in 16-month-old male zebrafish. (**a**–**d**) Representative photomicrographs Masson’s trichrome staining and fibrosis quantification with few fibrils lining the bile duct (arrowhead) and tumor-like cells (black stars) in *beclin1^+/−^*. (**e**–**h**) Representative photomicrographs of PAS staining with sufficient glycogen in WT and *atg7^+/−^* and sever glycogen reduction in *beclin1^+/−^*, notice the necrotic development around bile ducts (black stars). (**i**–**l**) ORO stain and lipid quantification showing hepatocytes hypertrophy with lipid accumulation and fatty clouds in *beclin1^+/−^* zebrafish. Scale magnification is shown in pictures and data are expressed as mean ± SD. * *p* < 0.05, ** *p* < 0.01, and *** *p* < 0.001. CV: Central Vein; BD: Bile Duct; NC: Necrosis.

To examine whether cirrhosis (fibrosis) happened in the hepatic tissue of *beclin1^+/−^*, liver sections were stained with Masson’s Trichrome, where the collagen fibers were stained with blue color. No cirrhosis was observed in the livers of WT and *atg7^+/−^* zebrafish at 12 and 16 month-old. Interestingly, *beclin1^+/−^* appeared also without chronic cirrhosis, and few fibrils surrounded the developing necrosis or lined the bile ducts (Figure 3Aa–c,Ba–c). The stained sections were scanned with a Pannoramic MIDI scanner to perform images quantification and analysis. The degrees of liver fibrosis were significantly increased in *beclin1^+/−^* zebrafish compared with the WT (Figure 3Ad,Bd).

To detect hepatic metabolic equilibrium, liver glycogen was stained with Periodic Acid-Schiff (PAS). Here, hepatocytes of the WT and *atg7^+/−^* contained an adequate amount of glycogen at 12-month-old as well as at 16-month-old (Figure 3Ae–g,Be–g), while *beclin1^+/−^* hepatocytes showed a severe reduction in the glycogen contents (Figure 3Ag,Bg). Moreover, Oil Red O (ORO) staining showed remarkable hypertrophy of hepatocytes with fatty changes and cloudy swellings in the liver tissue of *beclin1^+/−^* compared to WT and *atg7^+/−^* (Figure 3Ai–k,Bi–k). A significant decrease in hepatocyte glycogen and increase in hepatocyte lipids were detected and calculated in *beclin1^+/−^* compared to WT and *atg7^+/−^* zebrafish (Figure 3Ah,Al,Bh,Bl).

### 2.4. Beclin1^+/−^ Affects Hepatic Glucose/Lipid Flux on the Genetic Level

**Figure 4:**
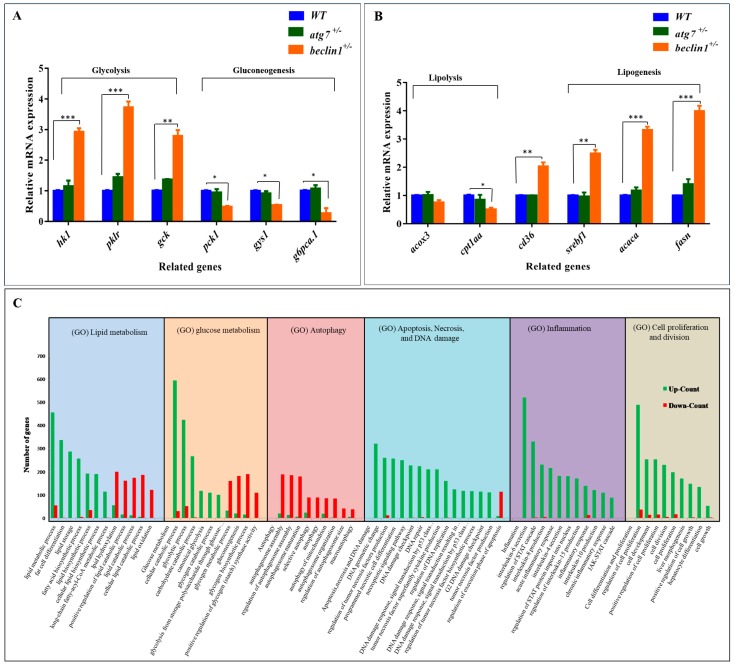
Effects of *beclin1* heterozygosity on the hepatic glucose/lipid flux at the genetic level. Expressions of mRNA in 16-month-old zebrafish evaluated by qRT-PCR for glycolysis and gluconeogenesis related genes (**A**) and lipolysis and lipogenesis related genes (**B**) indicating the disturbance of glycogen and lipid metabolism. (**C**) Gene Ontology (GO) analysis of differential expression genes (DEGs) in the liver between *beclin1^+/−^* and WT. The *y*-axis represents the number of genes of the biological processes in the *x*-axis. The numbers of upregulated DEGs (green bars) and downregulated DEGs (red bars) are shown. Data are expressed as mean ± SD. * *p* < 0.05, ** *p* < 0.01, and *** *p* < 0.001.

During liver tumorigenesis, the glycolysis pathway achieves the demands of cellular proliferation and energy [45]. Herein, mRNA expression of genes involved in glycolysis, including *hk1*, *pklr*, and *gck*, was upregulated in *beclin1^+/−^*. On the other side, mRNA expression of *pck1, gys1,* and *g6pca.1* genes that are involved in gluconeogenesis was downregulated (Figure 4A), and this confirms the former results of PAS staining. Alongside this, the lipogenesis pathway is frequently upregulated in human liver tumors [46]. Accordingly, mRNA expression of genes involved in lipogenesis including *srepf1*, *acaca*, and *fasn* was obviously induced in *beclin1^+/−^* liver compared with WT and *atg7^+/−^* siblings. On the other hand, mRNA expression of genes involved in lipolysis, including *acox3* and *cpt1aa*, was slightly declined in *beclin1* heterozygotes. However, ***cd36*** (fatty acid translocase) that controls adipocyte differentiation and accounts for long-chain fatty acids’ uptake by the hepatocytes [47], was highly induced in *beclin1^+/−^* (Figure 4B).

For more clarification, RNA transcriptome analysis was applied for *beclin1^+/−^* standardized with the WT. Here, Gene Ontology (GO) analysis of differential expression genes (DEGs) showed many enriched biological processes of glycol/lipid flux including upregulation of lipid biosynthetic process, fatty acids biosynthetic (Lipogenesis), and glycolytic process, as well as downregulation of genes counting for lipids catabolic process (lipolysis) and gluconeogenesis. Moreover, other biological processes could be monitored including the downregulation of autophagosome maturation and apoptosis, the enrichment of DEGs responsible for DNA damage, inflammation, and cellular proliferation (Figure 4C).

### 2.5. Unlike atg7^+/−^, Heterozygosity of beclin1 Suppresses Autophagy via Activating PI3K/AKT Pathway

**Figure 5:**
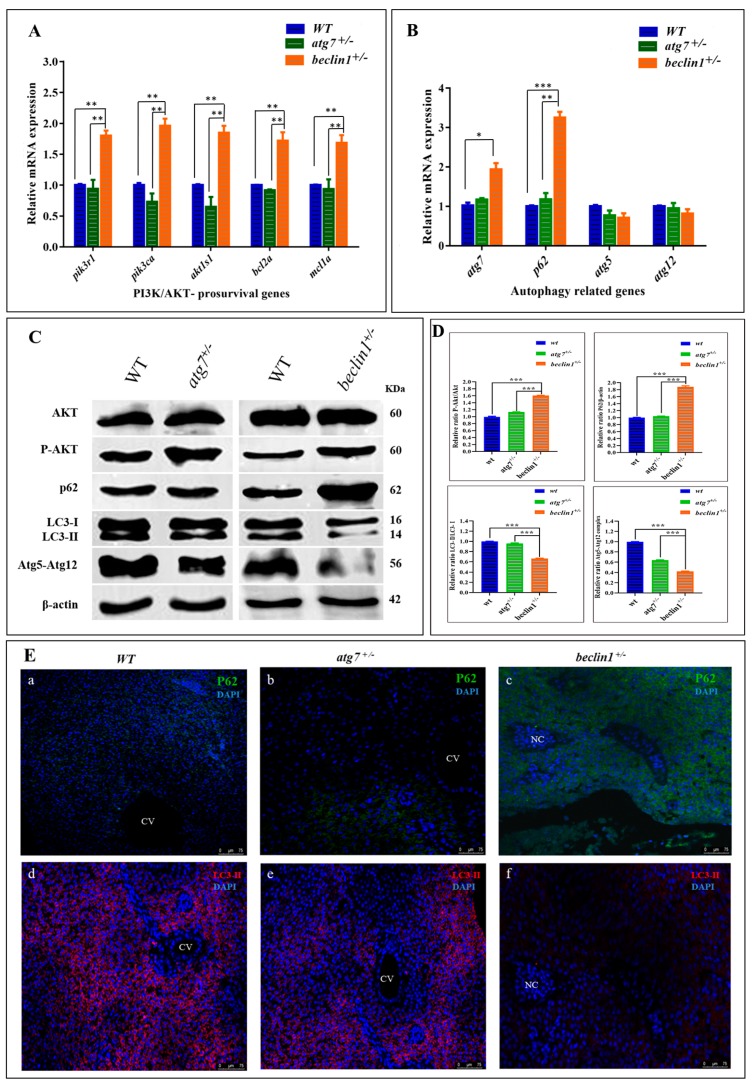
Dysregulated phosphoinositide-3-kinase (PI3K), the serine-threonine protein kinase (AKT) and autophagy pathways in the liver of *beclin1^+/−^* zebrafish. Expressions of mRNA in 16-month-old zebrafish evaluated by qRT-PCR for PI3K subunits and anti-apoptotic genes (**A**) and autophagy-related genes (**B**). (**C**) Representative immunoblots showing the relative protein expression of Akt, P-Akt, P62, LC3-I-II, Atg5-Atg12 conjugate. β-actin was used as vehicle control. (**D**) The ratio of P-Akt/Akt, P62/β-actin, LC3-II/LC3-I, and Atg5-atg12/β-actin quantified by NIH software Image J. (**E**) (**a**–**c**) Immunofluorescence of P62 showing protein increasing around the necrosis tissue of *beclin1^+/−^*.(**d**–**f**) Immunofluorescence of liver tissues probed against LC3-II revealed a severe reduction in *beclin1^+/−^*. CV: central vein; NC: Necrosis. Data expressed as mean ± SD. * *p* < 0.05, ** *p* < 0.01, and *** *p* < 0.001.

To assess the molecular mechanisms involved in hepatocellular malignancy and tumorigenesis of *beclin1*^+/−^, the mRNA expression of *akt1s1* and two phosphoinositide-3-kinase (PI3K) subunits including *pik3r1* and *pik3ca* as well as pro-survival (anti-apoptotic) genes including *bcl2a* and *mcl1a* were evaluated, which exhibited at higher levels in *beclin1^+/−^* compared with WT and *atg7^+/−^* littermates, indicating the activation influence of *beclin1^+/−^* on the PI3K/AKT pathway (Figure 5A). Furthermore, mRNA expression of autophagy-related genes, including *atg7*, *p62*, *atg5*, *atg12,* was evaluated in WT, *atg7^+/−^* and *beclin1*^+/−^ at 16-month-old. *Beclin1^+/−^* showed a significant increase in *atg7* expression indicating that there was an attempt to activate autophagy. However, there was still an obstacle to forming mature autophagosomes, and that is suggested by a higher expression of *p62* (Figure 5B). To evaluate the relationship between heterozygosity of *atg7* and *beclin1* and the PI3K/AKT/mTOR pathway, a Western blot was performed between the heterozygous strains and the WT within the same population. Herein, the protein levels of total Akt (Akt), phosphorylated Akt (p-Akt), P62, LC3I-II, and Atg5-Atg12 conjugated were evaluated as shown in Figure 5C. There was a significant increase in the ratio of p-Akt/Akt in *beclin1^+/−^*mutants compared with WT and *atg7^+/−^* individuals, whereas the level of total Akt was not affected. Moreover, *beclin1^+/−^* disturbed the canonical autophagy pathway by suppressing LC3-I lipidation, accelerating P62 accumulation, and inhibiting autophagosome formation by disturbing the *Atg5–*Atg12** conjugate (Figure 5C,D). For further verification, immunofluorescence (IF) was performed on liver sections from 16-month-old WT and both heterozygous *beclin1* and *atg7* at the same age. Here, *beclin1^+/−^* showed a significant increase in P62 protein around the necrotic area (Figure 5Ea–c) and a severe reduction in LC3-II (Figure 5Ed–f).

### 2.6. Induction of IL6/STAT3 Inflammatory Pathway and Downregulation of Apoptosis Pathway in beclin1^+/−^ Liver

**Figure 6:**
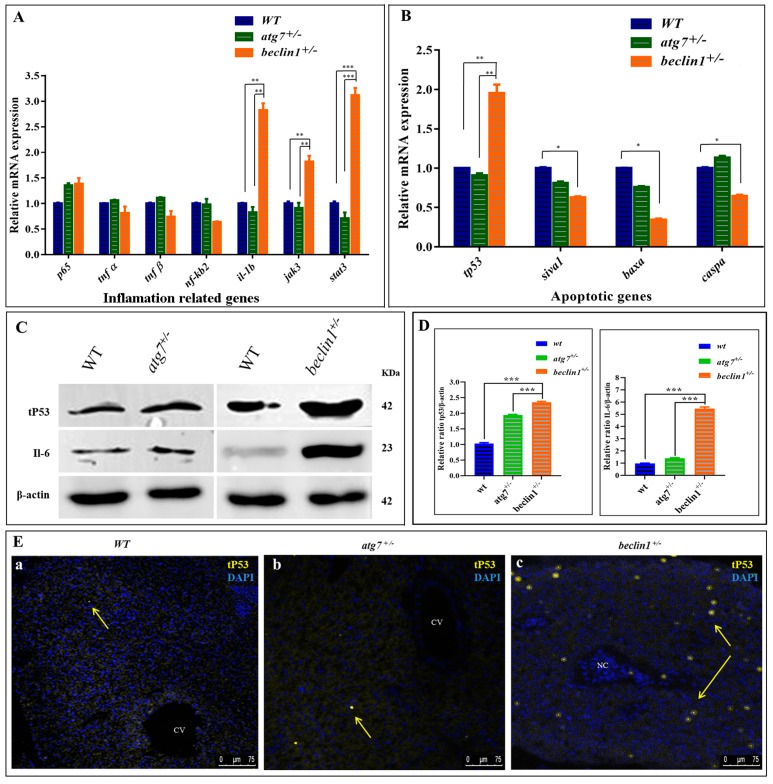
Abnormal apoptosis and inflammation response in *beclin1^+/−^* liver. Expressions of mRNA in 16-month-old zebrafish evaluated by qRT-PCR for inflammation-related genes (**A**) and apoptotic-related genes (**B**). (**C**) Representative immunoblots showed the relative protein expression of tP53, IL-6. β-actin was used as the vehicle control. (**D**) The ratio of tP53/β-actin and IL-6/β-actin evaluated by NIH software Image J. (**E**) Immunofluorescence of liver tissues probed against mutated tP53. The nuclei of the necrosis hepatocytes indicated the genomic instability and DNA damage in tumor cells (yellow arrows). CV: central vein; NC: Necrosis. Data expressed as mean ± SD. * *p* < 0.05, ** *p* < 0.01, and *** *p* < 0.001.

In the RNA-seq data, the downregulation of DEGs involved in apoptosis and upregulation of DEGs responsible for DNA damage, inflammation, and cellular proliferation were observed (Figure 4C). Herein, mRNA expression of genes involved in the two inflammatory pathways NF-kB and IL-6 was evaluated by qRT-PCR. It has been observed that heterozygosity of *beclin1* involved in the inflammatory response via activating *IL6/JAK/STAT3* pathway, as genes involved in this pathway (*il-1b*, *jak3*, and *stat3*), were upregulated, rather than genes involved in NF-kB pathway (*tnf α*, *tnf β*, *nf-κb2*) (Figure 6A). On the other side, mRNA expression of genes involved in pro-apoptosis, including *siva1*, *baxa*, and *caspa*, was significantly downregulated in *beclin1^+/−^* than in WT and *atg7^+/−^* siblings, but *tp53* mRNA was increased significantly (Figure 6B). The latter observations were confirmed by Western blotting that revealed the higher accumulation of tP53 as well as IL-6 proteins in the liver of *beclin1^+/−^* compared to WT or *atg7^+/−^* siblings (Figure 6C,D). Furthermore, to explore whether tP53 is a wild or mutated protein, the antibody against mutated tP53 was used and immunofluorescence was applied on the hepatic tissues, which showed that mutated tP53 was highly distributed in the necrotic tissue of *beclin1^+/−^* and located in the nuclei of the hepatocytes marking the damaged genomic DNA. This mutated tP53 that favors cancer cell survival and tumor progression is seldom distributed in WT and *atg7^+/−^* (Figure 6Ea–c).

### 2.7. Heterozygosity of beclin1 Enhances Hepatocellular Proliferation and Inflammation Probably Due to Apoptosis Suppression

**Figure 7:**
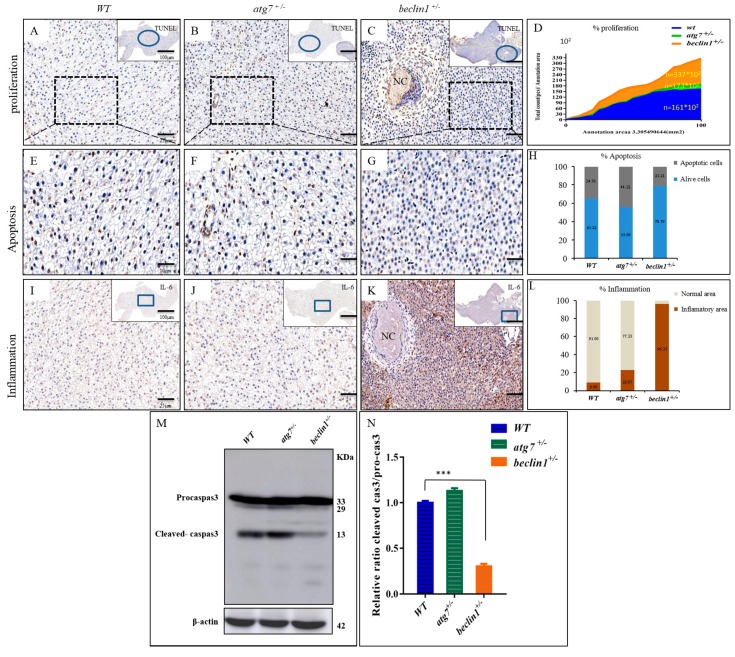
*Beclin1* heterozygosity induces cellular proliferation in the hepatic tissue with lower apoptosis and a higher risk of inflammation. (**A**–**C**) In situ detection of apoptotic cells by TUNEL staining by higher magnification of the blue circles. Brown apoptotic cells around necrosis (black arrow) are shown. (**D**) The mean cell number per unit area was quantified by NIH Image J. The results were normalized from three independent slides of the same area. (**E**–**G**) Magnified views of the boxed regions in (**A**–**C**). (**H**) Percentage of apoptotic cells according to E-G. The data are shown as the statistical results of three independent experiments, three fields scored per condition. (**I**–**K**) Immunohistochemistry assay of liver tissues blocked against IL-6 showed in higher magnification of the blue frames. (**L**) The signals in (**I**–**K**) were scanned with a Pannoramic MIDI scanner to perform image quantification and analysis. Results were summarized from three independent replicates and data were expressed by mean. (**M**–**N**) Western blot analysis and quantified protein expression showed that cleaved-caspase3 were downregulated in the *beclin1^+/−^* livers in comparison with the WT or *atg7^+/−^*. β-actin was used as an internal control. NC: Necrosis. * *p* < 0.05, ** *p* < 0.01, and *** < 0.001.

On the histological level, cellular apoptosis was detected by the TUNEL assay. The apoptotic cells (brown cells) were widely distributed in WT and *atg7^+/−^* zebrafish (Figure 7A,B,E,F), while they were mainly observed around the necrosis in *beclin1^+/−^* zebrafish (Figure 7C,G). All cells have been automatically counted in a fixed area (triplicate counting) that showed highly proliferative activity in the hepatocyte of *beclin1^+/−^* zebrafish (Figure 7D). The average percentages of apoptotic cells were obviously decreased in the liver of *beclin1^+/−^* zebrafish compared with that of WT or *atg7^+/−^* zebrafish (Figure 7H). To define the inflammatory role of the cytokines IL-6, immunohistochemistry of liver tissues revealed an aggressive accumulation of IL-6 protein in *beclin1^+/−^* unlike the depletion or almost absence of that protein in WT and *atg7^+/−^*, which is an obvious indication of chronic inflammation caused by the heterozygous distribution of *beclin1* (Figure 7I–K). In addition, the stained area density of IL-6 showed higher inflammation that covers more than 96% of the liver tissue of *beclin1^+/−^* compared to WT or *atg7^+/−^* (Figure 7L). Probably, the increased inflammation and uncontrolled proliferation in *beclin1^+/−^* liver is because of apoptosis limitation. For further clarification, Western blotting was performed and revealed that the cleaved-caspase3 was downregulated in *beclin1^+/−^* livers compared with WT or *atg7^+/−^* (Figure 7M,N).

## 3. Discussion

High carbohydrate diets can induce changes in the hepatic lipid metabolism that may lead to many malformations and disorders [48]. Autophagy plays important roles in maintaining homeostasis of nutrient metabolism in cultured fishes [7]. However, the potential molecular mechanisms are still largely unknown.

The PI3K/AKT/mTOR pathway is an intracellular signaling pathway mediating cell growth, proliferation, quiescence metabolism, survival, autophagy, and angiogenesis, and it has been reported to be involved in human cancer initiation [20,49,50]. Although the constitutive deletion of *atg7* is lethal at birth and conditional deletion of *atg7* in hepatocytes leads to benign liver tumors [51,52], these did not appear in our autophagy-defect model *atg7^+/−^* mutant zebrafish. But in *beclin1^+/−^* mutants, the glycolipid metabolism of the liver was disordered. The difference between *atg7* and *beclin1* may suggest the specific strategy of monoallelic deletion in the *beclin1* gene as a core component of the Vps34/Class III PI3K (PI3KC3) and Vps15/p150 complex that is sensitive to growth factor receptor signals [53].

Furthermore, in our autophagy-defect model *beclin1^+/−^*, the glycogen was consumed much more in the liver than WT or *atg7^+/−^* littermates and fat accumulation was significantly higher than fat consumption. This is because Akt requires glucose and relies on glycolysis to inhibit cell death [54]. The results showed *beclin1^+/−^* could alter the metabolism of the hepatocytes via increasing glycolysis and reducing lipolysis. A worse but interesting fact was that heterozygosity of *beclin1* induced solid hepatocellular necrosis through prolonged or chronic tumorigenesis, mainly in the male zebrafish. The results were similar to hepatocellular carcinoma (HCC) in humans, which has a male predominance worldwide [55,56]. However, the underlying mechanism is still obscure. This is because of the genetic basis of sexual dimorphism [57]. Interestingly, females with *beclin1^+/−^* exhibited spontaneous ovarian focal necrosis that affects the fertility and ceases ovulation.

Indeed, necrosis and inflammation are a doubled face coin as chronic inflammation is an important contributor to the increased risk of cancer development [56]. We revealed that *beclin1^+/−^* could induce inflammatory factors via activation of the *IL-6/Jak3/STAT3* inflammatory pathway that has been involved in other cancer studies [37,38,58]. Some other researches demonstrated that defective autophagy is the main cause of the involvement of *beclin1* in cancer [18,59]. However, we supposed that not only autophagy disturbance accounted for the hepatic necrosis but also other molecular mechanisms were involved in our zebrafish model. For example, it has been reported that cancer cells depend on the glycolysis process for energy production to facilitate cell survival and proliferation [54,60]. The lipogenic pathway is also an important hall marker of HCC that also resulted from the activation of the AKT pathway [61,62], and *cd36* has also been reported to increase in cancer [63]. Moreover, in our result, activation of the AKT pathway in *beclin1^+/−^* mutants led to the upregulation of mutated tP53 protein. Mutated tP53, unlike wild tP53 protein, can provide a suitable microenvironment for tumor cells’ survival and proliferation and that was confirmed in our study via a reduction in apoptosis, and/or the reinforcement of cell proliferation and inflammation [64].

The autophagy pathway was disturbed in *beclin1^+/−^* mutants which was determined by analyzing the expression of LC3-II and P62, the two markers used to monitor the autophagy flux [65,66]. However, here we determined that the other main ATG components of autophagy were well expressed by qRT-PCR, while autophagy impairment was due to the Atg5-Atg12 conjugate deficiency that affects autophagosomes formation observed by Western blotting. Briefly, a haplotype of the autophagy key gene *beclin1* could induce liver defects and lipid metabolism disorder and gradually develop into solid hepatocellular necrosis. The results on zebrafish *beclin1* functions could provide a clue to maintain homeostasis of nutrient metabolism in the cultured fishes.

## 4. Materials and Methods

### 4.1. Establishment of atg7 and beclin1 Mutant Lines and Calculation of Survival Rates

All experiments involving zebrafish were approved in compliance with the requirements of the IACUC of Huazhong Agricultural University (HZAUFI-2015-006, approved on 26 February 2016). The AB strain zebrafish was reared at the Huazhong Agriculture University, according to the establishes protocol [67]. All sgRNAs were designed using CRISPR RGEN Tools (http://www.rgenome.net). The linearized Cas9 plasmids were transcribed into mRNA using the T7 m MESSAGE Kit (Ambition, Austin, TX, USA) and gRNA was synthesized using a transcript Aid T7 High Yield Transcription Kit (Thermo Scientific, Waltham, MA, USA). Zebrafish eggs at the one-cell stage were co-injected with 20 pg target gRNA and 300 pg Cas9 mRNA. For genotyping, the following primers were used. *atg7-*F: 5’-AAATGCCACAGTCCTCCTC-3′, *atg7-*R: 5′-TGAGCCCAGCCTTTATTCT-3′ (399 bp), *beclin1*-F: 5’-GTATGCCATCAACCTCCTA-3’, and *beclin1*-R: 5’-AAAGTGAAGCACTGCGAAT-3′ (310 bp).

During the early development, phenotype and survival rates were regularly noted. At 3-month-old, genomic DNA was extracted from the tail regions of all males of the *atg*7^+/−^ and *beclin1*^+/−^ F2 generation. WT, heterozygous fishes of *atg7* and *beclin1* strains were divided into three groups (*n* = 50 for each) for survival rates’ assessment. The survival probability at any particular time (S_t_) was calculated and analyzed by the Kaplan–Meier plot [68].

### 4.2. Assessments of Liver Histology

For histological observations, mature males at 6, 12, and 16 month-old from each genotyped strain were dissected, and the livers were fixed in Bouin’s solution for 3 h at room temperature. Tissue sections of 5 μm thick were prepared using a microtome (Leica, Wetzlar, Germany). For light microscope analysis, histological sections were stained with hematoxylin and eosin (H&E). Masson’s trichrome staining, periodic acid-Schiff (PAS) and Oil Red O (ORO) were performed in the liver sections, as previously described [69].

### 4.3. RNA Extraction and Quantitative Real-Time PCR (qRT-PCR)

Total RNA was extracted from the livers of 6 adult male zebrafish for each sample. The RNA quality and concentration were evaluated by gel electrophoresis and NanoDrop 2000. Isolated RNA was reverse transcribed into cDNA by a PrimeScript TM RT reagent Kit with gDNA Eraser (Takara, Kyoto, Japan). Real-time PCR was performed on ABI-7500 real-time PCR machine (Applied Biosystems, Carlsbad, CA, USA) and β-actin was used to normalize the expression values [70]. The primers sequences used in real-time PCR are listed in Table S1.

### 4.4. Protein Extraction and Western Blotting

Homogenates of 6 adult livers from each strain at 16-month-old were used for total protein extraction and kept at −80 °C. Western blot was performed according to the previous reports [71]. Equal amount protein of each sample was separated in 10% SDS-PAGE gel, transferred onto Polyvinylidene Fluoride (PVDF) membranes (Millipore, Burlington, MA, USA) and blotted with primary antibodies, rabbit Akt (Cell Signaling, 9272S, Boston, MA, USA), rabbit P-Akt (Cell Signaling, 4060S, Boston, MA, USA), rabbit SQSTM1/P62 (MBL, PM045, Woburn, MA, USA), rabbit LC3A/B (Cell Signaling, 4108, Boston, MA, USA), rabbit tP53 (GeneTex, GTX128135, Irvine, CA, USA), rabbit IL-6 (Abcam, ab208113, Cambridge, UK), rabbit Atg5-Atg12 conjugate (Novus biology, NB110-53818SS, Perth, WA, AUS), recombinant anti-Caspase-3 p12 antibody (EPR16888) (Abcam, ab179517, Cambridge, UK) and β-actin (Cell Signaling, 4967S, Boston, MA, USA), separately. Blots were probed with horseradish peroxidase (HRP)-conjugated secondary antibody and visualized using enhanced chemiluminescence reagent (ECL) ((GE Healthcare Bio-Sciences Corp (formerly Amersham Pharmacia Biotech), Piscataway, NJ, USA)) Western blotting detection reagents. NIH software Image J (National Institutes of Health, Rockville, MD, USA) was applied for blot scanning and protein quantifications.

### 4.5. Immunohistochemistry, TUNEL Assay, and Immunofluorescence

For immunohistochemistry, fixed paraffin sections of 16-month-old zebrafish strains were used. Sections were dewaxed and hydrated, followed by 10 min of antigen retrieval in sodium citrate buffer (pH 6.0) at 100 °C. Slides were treated with 0.3% H_2_O_2_ for 10 min to remove endogenous peroxidase, then blocked with 5% bovine serum albumin (BSA) in phosphate-buffered saline tween 20 (PBST) for 1 h at room temperature. Sections were incubated with the first antibody rabbit IL-6 (Abcam, ab208113, Cambridge, UK) (1:100) at 4 °C overnight. After washing 3 times with PBST, the sections were incubated with 50–100 µL HRP secondary antibody for 1 h at room temperature followed by staining 3,3′-diaminobenzidine (DAB) substrate and counterstained with hematoxylin for nuclear differentiation. Cell death in the liver tissues was detected by using a TUNEL assay kit. The methodology was previously mentioned elsewhere [72].

Immunofluorescence was performed as the following steps. After antigen retrieval by sodium citrate buffer, slides were blocked with PBS with TritonX-100 (PBT) containing 5% BSA and for 1 h at 37 °C. Sections were incubated with the primary antibodies including, rabbit LC3B (Abcam, ab483940, Cambridge, UK) (1:200), rabbit SQSTM1/P62 (MBL, PM045, Woburn, MA, USA), (1:500), rabbit Anti-Mutant P53 antibody (Y5) (Abcam, ab32049, Cambridge, UK) (1:100) at 4 °C overnight. After washing three times in PBST, slides were incubated with fluorescein-conjugated secondary antibodies for 1 h at room temperature. Nuclei were stained with 4′, 6-diamidino-2-phenylindole (DAPI). Sections were analyzed by fluorescence microscopy (Leica, Wetzlar, Germany) using an Axio Vision image capture system (Carl Zeiss, Oberkochen, Germany).

### 4.6. Image Acquisition and Digital Image Analysis

The livers embedded in paraffin blocks were sectioned at 5 μm. After histological assessments, images were captured by Case Viewer software. Images quantification and analysis were performed by the 3D Histech Quant Center using a tissue slice scanner model Pannoramic MIDI (3DHISTECH, Budapest, Hungary). The semi-quantitative analysis of the staining intensity was carried out under the lens of the scanner and the tissue information form a file using the Pannoramic viewer software and could be amplified by 1-400 at any location. The magnification power covers different areas (10× = 100 µm, 100× = 10 µm, 40× = 25 µm, 400× = 2.5 µm). The degree of liver fibrosis, hepatocyte glycogen, hepatocyte lipids, and inflammatory area were estimated in 10 different randomly selected 100× fields, following the manufacturer’s recommendations [73].

### 4.7. Transcriptomic Sequencing (RNA-Seq) and Gene Ontology

Total RNAs were isolated from the livers of six wild-type and six *beclin1^+/−^* zebrafish (male, 12-month-old), respectively. Sequencing and analysis were performed in the Annoroad Biotechnology Company (Zhejiang, China) following the manufacturer’s protocol. The purity and concentration of RNA were checked using the spectrophotometer and RNA Nano 6000 Assay Kit of the Bioanalyzer 2100 system (Agilent Technologies, CA, USA) respectively. Only RNA with OD 260/280 ≥ 1.9 and RNA integrity number ≥7 were selected for the subsequent experiments. For RNA library preparation, a total amount of 2 μg RNA per sample was used as input material for the RNA sample preparations. Sequencing libraries were generated using the NEBNext^®^ Ultra™ RNA Library Prep Kit for Illumina^®^ (#E7530L, NEB, Ipswich, MA, USA) following the manufacturer’s recommendations [74]. In this study, we selected differentially expressed genes based on fold change >2 (statistical power > 0.8) and *p*-value < 0.05 that were used for further analysis of the all upregulated and downregulated gene for gene ontology (GO) of different biological processes.

### 4.8. Statistical Analysis and Graphs Preparation

Data are presented as mean ± SD from three dependent experiments. Statistical analysis was performed using SPSS and the data were analyzed by Student’s *t*-test when comparing two groups and one-way ANOVA for comparison more than two groups (* *p* < 0.05; ** *p* < 0.01; *** *p* < 0.001). Graphics and plots were designed using GraphPad Prism 8 software package (GraphPad Software, La Jolla, USA).

## Supplementary Materials

The following are available online at https://www.mdpi.com/1422-0067/21/4/1533/s1.
