# Supplementary material for: Strategy of Hepatic Metabolic Defects Induced by beclin1 Heterozygosity in Adult Zebrafish
Source: Int J Mol Sci. 2020 Feb 24;21(4):1533. doi: 10.3390/ijms21041533 (PMC7073209; doi:10.3390/ijms21041533)
Supplement: Supplementary file 1 [file ijms-21-01533-s001.pdf]

Supplementary Materials

Table S1 The primers sequences used in real-time PCR.

Gene	Name	Sequences (5′–3′)	Accession (Gene ID)
hk1	hexokinase 1	F: 5' ACTTTGGGTGCAATCCTGAC 3'<br>R: 5' AGACGACGCACTGTTTTGTG 3'	NM_213252
pklr	pyruvate kinase L/R	F: 5' TCCTGGAGCATCTGTGTCT 3'<br>R:5' GTCTGGCGATGTTCATTCCT 3'	NM_201289
gck	glucokinase	F:5' GCTGTGAAGTCGGCATGATA 3'<br>R:5' CTTCAACCAGCTCCACCTTAC 3'	NM_001045385
pck1	Phosphoenolpyruvate carboxykinase 1	F:5' ATCACGCATCGCTAAAGAGG 3'<br>R:5' CCGCTGCGAAATACTTCTTC 3'	NM_214751
gys1	glycogen synthase 1	F:5' GCAGCTCAG TGTGACGAACC 3'<br>R:5' GGTCCCCTGCTTCCTTATCC 3'	NM_201180
g6pca.1	glucose-6-phosphatase a, catalytic subunit, tandem duplicate 1	F:5' TCACAGCGTTGCTTTCAATC 3'<br>R:5' AACCCAGAAACATCCACAGC 3'	NM_001003512
acox3	Acyl-CoA oxidase 3	F: 5′-AAGGACATCGAGCGAATGAT 3′<br>R:5′-CTATGAAAGAGTGGAGGCCG 3′	NM_213147
Cpt1aa	Carnitine palmitoyltransferase1Aa	F: 5′-ACTCTCGATGGACCCTGTGA-3′<br>R:5′-CTGGATGAAGGCATCTGGAC-3′	NM_001044854
cd36	Thrombospondin receptor	F: 5′AGGCCACTGTGAACCTGAAG 3′<br>R: 5′AAGTTGGGGTTCATTCCGAC 3′	NM_001002363
srebf1	Sterol regulatory element binding protein1	F: 5′CATCCACATGGCTCTGAGTG 3′<br>R: 5′CTCATCCACAAAGAAGCGGT 3′	NM_001105129
acaca	acetyl-CoA carboxylase alpha	F: 5′ GCATAGGGCAGGTTTTACCA 3′<br>R:5′GCCATCATACGAGAGCAACA3′	NM_001271308
fasn	Fatty acid synthase	F: 5′GAGAAAGCTTGCCAAACAGG3′<br>R:5′GAGAAAGCTTGCCAAACAGG3′	XM_009306806
pik3r1	phosphoinositide-3-kinase, regulatory subunit 1	F:5' ACATGGCTCTGCAAGATGCT3'<br>R:5' GGAGGCATCTCGGACCAAAA3'	NM_001281844
pik3ca	phosphatidylinositol-4,5-bisphosphate 3-kinase, catalytic subunit alpha	F:5' CGCAATGAGAGGATGAGCGA3'<br>R:5' ACGCTGTCACGATGGAACAA 3'	XM_009306176
akt1s1	AKT1 substrate 1 (proline-rich)	F: 5' TCGGCAGGTGTCTTCTCAAT 3'<br>R:5' ACCCATTGCCATACCACGAG 3'	XM_687419
bcl2a	BCL2 apoptosis regulator a	F: 5' GATTTACCAACGCGAATTTGAGGA 3'<br>R: 5' GTCCCACCAAACTCGAAGAATG 3'	NM_001030253
mcl1a	MCL1 apoptosis regulator, BCL2 family member a	F: 5' AGCTCTTTAGCGATGGCACCACAA 3'<br>R:5' AGCCATCCCATGCTTTGTTTCTGA 3'	NM_131599
atg7	autophagy related 7 homolog	F:5'ACGGTGATGCTGTTGGTCTG 3'<br>R: 5' TTTGTCGGTGGATTTGAAGG 3'	XM_021479676
p62	sequestosome 1	F:5' TGGTGCTACTGCCTCTTCTCA 3'<br>R:5' GGGTTACTTTGGTCCGCTTT 3'	NM_001312913
atg5	autophagy related 5 homolog	F:5' TGGAGTATCCCACCGAAGA3'<br>R: 5' TGCCGTGAATCATAACCTG 3'	NM_205618
atg12	autophagy related 12 homolog	F:5' TCATCTCACGCTTCCTCAA 3'<br>R: 5' TCACTTCCGAAACACTCAAA 3'	NM_001246200
p65	RELA	F:5' GGAGAAGCGCAAGAGAACTGA 3'<br>R:5' CGTAGGGAATGGCCGTCTTT 3'	NM_001001839
tnf α	Tumor necrosis factor a	F:5' AGACCTTAGACTGGAGAGATGAC 3'<br>R:5' CAAAGACACCTGGCTGTAGAC 3'	NM_212859
tnf β	tumor necrosis factor b	F: 5' TCAGAAACCCAACAGAGAACATC 3'<br>R:5' ACCCATTTCAGCGATTGTCC 3'	NM_001024447
nf-κb2	kappaB kinase/NF-kappaB cascade	F: 5' GAAGCATTCAGGCTCGGTGA 3'<br>R: 5' CAGGTCTGTCGGTCCCTTTC 3'	NM_001001840
il-1b	interleukin 1, beta	F: 5' TGGCGAACGTCATCCAAG 3'<br>R:5' GGAGCACTGGGCGACGCATA3'	NM_212844
jak3	Janus kinase 3 (a protein tyrosine kinase, leukocyte)	F: 5' AACAGAGCGAGCAGCAGAGAG 3'<br>R: 5' GTGTGACCACCCTTCCTTCC 3'	XM_002663087
stat3	signal transducer and activator of transcription 3 (acute-phase response factor)	F: 5'ACAGCAGGATGGCCAGGTTGC 3'<br>R: 5'TCTGTCTGGTGGCTGCTGCCT 3'	NM_131479
tp53	tumor protein p53	F: 5′ GCAGTCTGGCACAGCAAAATCTGT 3′<br>R: 5′ TCAGCCACATGCTCGGACTTCTTA 3′	NM_131327
siva1	apoptosis-inducing factor	F: 5 ' CCGCTACCGACAGGAGATCTACGA3′<br>R: 5′ GGTGTGGAGCGCGCTCTGTGCAGT 3′	NM_001327928
baxa	BCL2 associated X, apoptosis regulator a	F: 5 GTCGGAGTTTTCCTCGCTGGAGTT 3′<br>R: 5′ AGGCGGTTTCACCTCTCAATGCTT 3′	NM_131562
caspa	caspase a	F: 5′ GACGGTGAGCCTGATGAGCCAA 3′<br>R: 5′ CCTGAACAGTTCCTCGATGTGA 3′	NM_131505
β-actin	actin, beta 1	F: 5' TCCCTGTATGCCTCTGGTCGT 3'<br>R:5'AAGCTGTAGCCTCTCTCGGTC 3'	NM_131031
